# Antidiabetic activity of the chemical constituents of Combretum dolichopetalum root in mice

**DOI:** 10.17179/excli2016-252

**Published:** 2016-04-25

**Authors:** Philip F. Uzor, Patience O. Osadebe

**Affiliations:** 1 Department of Pharmaceutical and Medicinal Chemistry, Faculty of Pharmaceutical Sciences, University of Nigeria, Nsukka, 410001, Enugu State, Nigeria

**Keywords:** antidiabetic constituents, arjunolic acid, Combretum dolichopetalum, combretaceae, echinuline, ellagic acid

## Abstract

The root of* Combretum dolichopetalum* (Combreatacea) is used in ethnomedicine for the management of diabetes mellitus. Though some compounds have been isolated from it, the antidiabetic principles have not been identified. The present study was designed to evaluate the chemical constituents from the root of *C. dolichopetalum* with a view to identifying the antidiabetic principles. The constituents include the alkaloids, echinulin (**1**) and arestrictin B (**2**), the terpenoids, arjunolic acid (**3**) and 4'-dihydrophaseic acid (**4**) as well as the phenolic acids, ellagic acid (**5**) and 3, 4, 3'-tri-O-methylellagic acid (**6**). Twenty eight mice (in seven groups, n = 4) were made diabetic using alloxan monohydrate (*i.p*., 120 mg/kg) and treated orally with either the vehicle (control group), any of the constituents or glibenclamide (standard drug). The fasting blood glucose of the diabetic animals was monitored for nine hours. Results showed that all the chemical constituents (**1**-**6**) exhibited significant (*p* < 0.05) antidiabetic activity comparable to glibenclamide. The alkaloids exhibited the most profound antidiabetic activity. The present study has thus identified the antidiabetic principles of *C. dolichopetalum* root as echinulin, arestrictin B, arjunolic acid, 4'-dihydrophaseic acid, ellagic acid and 3, 4, 3'-tri-O-methylellagic acid. The study has further validated the ethnomedicinal use of the root of *C.* *dolichopetalum* in diabetes.

## Introduction

Diabetes mellitus is a chronic metabolic disorder which is characterized by hyperglycemia and long term complications such as retinopathy, nephropathy, neuropathy, and angiopathy. Diabetes remains one of the leading causes of death worldwide. The 2015 estimate by the International Diabetes Federation (IDF) shows that 1 in 11 adults have diabetes (415 million) and the figure is projected to rise to 1 adult in 10 (642 million) in 2040. The estimate also shows that diabetes caused 5.0 million deaths implying that every six seconds a person dies from diabetes. This metabolic disorder caused at least USD 673 billion, representing 12 % of global health expenditure (IDF, 2015[8]). The increasing worldwide incidence of diabetes constitutes a significant impact on the health, quality of life, and life expectancy of patients, as well as on the global public health care system. 

In conventional practice, management of type 1 diabetes is by exogenous insulin while type 2 is by oral agents including the sulfonylureas, biguanides, α-glucosidase inhibitors, glitazones, gliptins, and the SGLT 2 inhibitors, which are used as monotherapy or in combination to achieve better glycemic regulation since they have different modes of action. Despite the existing pharmacotherapy, it is still difficult to attain adequate glycemic control amongst many diabetic patients due to the progressive decline in β-cell function (Wallace and Matthews, 2000[25]). Moreover, many of these oral antidiabetic agents have a number of serious adverse effects (Pepato et al., 2005[17]). The low efficacy and the adverse effects of some of the conventional antidiabetic agents together with the increasing global incidence of diabetes necessitate the urgent search for more potent and safer alternatives from natural sources such as medicinal plants. 

**Figure 1:**
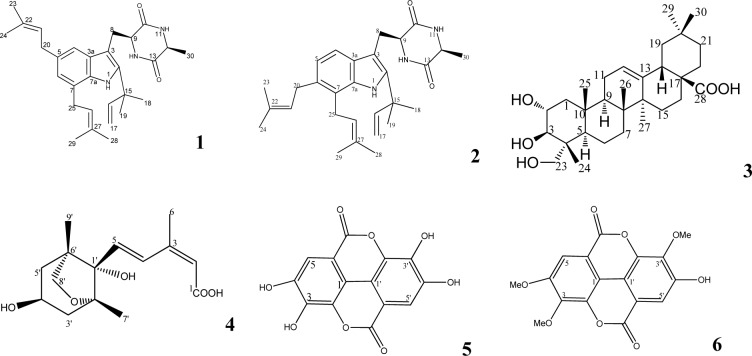
Isolated compounds from *C. dolichopetalum* root

As part of our on-going search for antidiabetic agents from natural sources (Osadebe et al., 2014[16]; Uzor et al., 2014[24]), we have furthered our investigation on the root of *Combretum dolichopetalum* (Combretaceae) Engl. and Diels. The plant is a scandent shrub or forest liana. The root extract is used in traditional medicine for dysentery, indigestion and in the management of diabetes (Uzor et al., 2014[24]). The antisecretory (Asuzu and Onu, 1990[1]), anti-hepatotoxic (Udem et al., 1997[22]) and trypanocidal (Udem et al., 1996[21]) activities of this plant have been reported. We have recently investigated the antidiabetic activity of the root extract and fractions of this plant on diabetic animals and established that the antidiabetic fractions contain steroids, terpenes, alkaloids and flavonoids (Uzor et al., 2014[24]). Additionally, our more recent chemical investigation led to the isolation of six known metabolites (Figure 1(Fig. 1)) from the root of the plant, including the alkaloids echinulin (**1**) and arestrictin B (**2**), terpenoids, arjunolic acid (**3**) and 4'-dihydrophaseic acid (**4**), as well as phenolic acids ellagic acid (**5**) and 3, 4, 3'-tri-O-methylellagic acid (**6**) (Uzor et al., 2015[23]). These compounds were investigated for their cytotoxic potentials using the L5178Ymouse lymphoma cell lines but their antidiabetic potentials have not been assessed. Thus the antidiabetic constituents of the root of this plant have not been identified. The current study was therefore designed to evaluate the antidiabetic activity of the isolated constituents from the root of this plant. To the best of our knowledge, this is the first report of antidiabetic compounds from *C. dolichopetalum* root.

## Experimental

### Chemicals

Alloxan monohydrate was purchased from Sigma-Aldrich while glibenclamide was purchased from NGC. All other chemicals were of analytical grade.

### Plant material

Roots of *Combretum dolichopetalum *were collected in Nsukka, Nigeria, in April 2014 and identified by Mr. A. Ozioko, a taxonomist with the International Center for Ethnomedicine and Drug Development (InterCEDD), Nsukka. A voucher specimen (InterCEDD/853) is deposited at the Herbarium of the Department of Pharmaceutical and Medicinal Chemistry, University of Nigeria, Nsukka. 

### Extraction and isolation 

The procedures for the isolation of the constituents of *C. dolichopetalum* root to obtain **1-6 **have been recently described (Uzor et al., 2015[23]). Compounds **1** and **2 **(or **1**/**2**), two isomeric indole-containing diketopiperazine alkaloids, were obtained as a mixture (56:54). They were thus evaluated as one sample. 

### Animals

Swiss albino mice (18.0-24.2 g) of either sex were used for the studies. The animals were kept in standard polypropylene cages at room temperature and 60-65 % relative humidity during the experimental work with 12 h day: 12 h night cycle. They were fed with normal laboratory diet and allowed to drink water *ad libitum*. The experimental protocols followed the guidelines of the Ethics Committee of the University of Nigeria and the European Community guidelines (EEC Directive of 1986; 86/609/EEC) (EEC, 1986[3]).

### Antidiabetic study 

Twenty eight (28) albino mice were fasted for 12 h prior to induction of hyperglycemia. Diabetes was induced using freshly prepared solution of alloxan monohydrate (*i.p*. at 120 mg/kg body weight) as previously reported (Uzor et al., 2014[24]). After the induction, the diabetic animals were randomly divided into seven groups (n = 4) and treated orally according to the following protocols: 

Group A: alloxan + 2 mL/kg of vehicle

Group B: alloxan +20 mg/kg of **1**/**2**

Group C: alloxan + 20 mg/kg of **3**

Group D: alloxan + 10 mg/kg of **4**

Group E: alloxan + 20 mg/kg of **5**

Group F: alloxan + 20 mg/kg of **6**

Group G: alloxan + 0.2 mg/kg of glibenclamide 

Blood sample was withdrawn from the tail vein and fasting blood glucose (FBG) was measured with a glucometer (Accu-chek®, Roche) at 0, 1, 3, 6 and 9 h. Mice model was used since the compounds were limited in quantity. 

### Statistical analysis

The data obtained were analyzed using a statistical software (SPSS version 21) and results expressed as mean ± SEM. The means were subjected to one way analysis of variance (ANOVA) for determining the significant difference. The significance between the various groups and the control was analyzed further by *post hoc* Dunnet's test (2-sided). Group mean was considered to be significantly different from that of the control group at *p* < 0.05.

## Results

The results of the antidiabetic activities of the isolated chemical constituents are shown in Table 1(Tab. 1). All the constituents (**1**-**6**) exhibited significant (*p* < 0.05) reduction of the FBG of the diabetic mice after 3 h but the effect of the alkaloids, (**1**/**2)**, was most pronounced and sustained. The antidiabetic effect of the alkaloids was maximal at 3 h after administration (53.40 % reduction of FBG). Among the constituents, **1**/**2** was followed in antidiabetic potency by **5** (23.7 % maximal reduction in 9 h), **6** (22.7 % reduction in 3 h), **4** (17.91 % maximal reduction of FBG in 6 h), and then by **3** (17.27 % reduction in 3 h). Even at 10 mg/kg, **4 **exhibited significant antidiabetic activity by lowering the FBG (17.91 %) of the diabetic mice. Overall, the effect of the compounds was comparable to that of glibenclamide (reduction of 48.72 % in 9 h).

## Discussion

The present study has shown that the compounds from the root of *C. dolichopetalum* possess antidiabetic activity. The present observation validates the traditional use of the plant in diabetes. Other plant species from Combretaceae have been known to contain diverse chemical constituents such as ellagic acid, gallic acid, ellagitannins and gallotannins; they have been also known for a long time to show pharmacological effects like antioxidant, antibacterial and antidiabetic activities (Sabu and Kuttan, 2002[18]). Previous report shows that echinuline (**1)** exhibits moderate protein tyrosine phosphatase 1B (PTP1B) inhibitory activity (Sohn et al., 2013[19]) which suggests the possible antidiabetic activity of **1**. The biological activity of arestrictin B (**2**) was not evaluated in a study where it was first isolated in a pure form from xerophilic fungi (Itabashi et al., 2006[9]). Thus it is possible that one or both of the two isomeric alkaloids possess antidiabetic activity or they could be acting synergistically. Further studies on the separation of the alkaloids and evaluation of their individual antidiabetic activities are envisaged. These two compounds have been isolated singly especially from fungal sources but they were reported, for the first time, as having occurred together in *C. dolichopetalum* root (Uzor et al., 2015[23]).

Similar to the alkaloids above, our findings showed that arjunolic acid (**3)** was one of the antidiabetic constituents of *C. dolichopetalum*. The triterpenoid, **3**, is also possibly acting synergistically with other bioactive constituents. Previous reports support our present observation. Compound **3 **was isolated from* Lagerstroemia speciosa* leaves and tested for its alpha glucosidase activity using yeast alpha glucosidase enzyme. It was shown that **3** inhibited the enzyme with IC_50_ of 18.63 ± 0.32 µg/mL while the standard drugs, acarbose and voglibose, showed no inhibition (Hou et al., 2009[7]). Other studies have also supported the beneficial role of **3** in diabetes (Ghosh and Sil, 2013[4]; Manna et al., 2009[14][15]; Manna and Sil, 2012[13]). 

The results of the present study have shown that dihydrophaseic acid (**4)**, a derivative of *S(+)*-abscisic acid (ABA) (Hirai et al., 2003[6]) exhibits significant (p < 0.05) antidiabetic property. ABA, a plant hormone, has been shown to exhibit antidiabetic effect through peroxisome proliferator-activated receptor gamma (PPAR γ) activation** (**Guri et al., 2010[5]). It is therefore possible that **4 **exhibits its antidiabetic activity through PPAR γ activation similar to its precursor, ABA. The present study is probably the first report of the antidiabetic activity of this ABA metabolite. However, one study shows that **4** exhibited some biological activities such as cytotoxicity, lipid peroxidation inhibition effect, mild hydroxyl radical scavenging activity, and DNA protective effect (Lin et al., 2014[11]). 

We have also observed that ellagic acid (**5**) was one of the bioactive antidiabetic principles of *C. dolichopetalum* root. Previous study has demonstrated the antidiabetic activity of **5** *in vivo* (Malini et al., 2011[12]). Moreover, **5** has been widely reported as an inhibitor of aldose reductase enzyme and inhibitor of the formation of advanced glycation end products (Kim et al., 2008[10]). Aldose reductase inhibitors are an attractive pharmacological target for the treatment of diabetic complications. Compound **6**, which is a methylated derivative of **5** also showed *in vivo* antidiabetic activity in the present study. Earlier studies showed the α-glucosidase inhibitory effect of the methylated derivatives of **5** (Tabopda et al., 2008[20]). Furthermore, methylated derivatives of **5** are known to play some roles in antidiabetic activities of plants. While **6** showed glucose transport stimulatory activity, **5** did not show such activity (Bai et al., 2008[2]). This suggests that methylation of the OH group of **5** is necessary for antidiabetic activity. 

## Conclusion

The present study has shown that all the isolated constituents of *C. dolichopetalum* root including the alkaloids (echinulin and arestrictin B), arjunolic acid, 4'-dihydrophaseic acid, ellagic acid and as 3, 4, 3'-tri-O-methylellagic acid were identified as the major antidiabetic active principles of the plant. It is possible that these metabolites exert synergistic effect in their antidiabetic activities. The study has further validated the ethnomedicinal use of the root of *C.* *dolichopetalum* in the management of diabetes. 

## Acknowledgement

The authors are grateful to the Institute of Pharmaceutical Biology and Biotechnology, Universität Dusseldorf for HPLC and NMR measurement of the isolated compounds. They are also grateful to Mr. O. Ezeugwu for assistance in sourcing the plant materials and Mr. A. Ozioko for the identification of the plant. 

## Conflict of interest

No conflict of interest is associated with this work.

## Figures and Tables

**Table 1:**
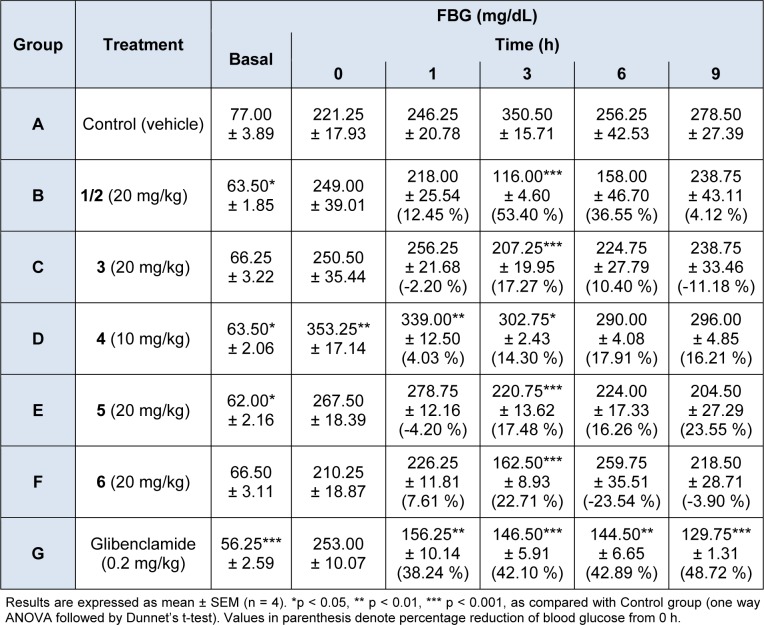
Effect of the chemical constituents from *C. dolichopetalum* root on the FBG of alloxan-induced diabetic mice
